# Crystal structure of (1*S*,2*S*,5*R*)-5-acetyl­amino-4-oxo-2,3-diphenyl-1,3-thia­zinan-1-ium-1-olate

**DOI:** 10.1107/S2056989017012488

**Published:** 2017-09-05

**Authors:** Hemant P. Yennawar, Duncan J. Noble, Lee J. Silverberg

**Affiliations:** a Department of Biochemistry and Molecular Biology, Pennsylvania State University, University Park, PA 16802, USA; b Pennsylvania State University, Schuylkill Campus, 200 University Drive, Schuylkill Haven, PA 17972, USA

**Keywords:** 1,3-thia­zin-4-one, twisted half-chair pucker, N—H⋯O and C—H⋯O inter­actions, crystal structure

## Abstract

The asymmetric unit of the enanti­omerically pure title compound, C_18_H_18_N_2_O_3_S, comprises two independent mol­ecules (*A* and *B*) having almost identical conformations. When overlayed, the alignment–r.m.s. deviation value is 0.30 Å. The six-membered heterocycle has a twisted half-chair conformation in both mol­ecules. The O atom on the S atom of the ring is pseudo-axial on the thia­zine ring and *trans* to both a phenyl group substituent and the acetamide group in each case. The two benzene rings in each mol­ecule are almost orthogonal to each other, with inter­planar dihedral angles of 83.79 (17) and 86.95 (16)°. The acetamide group is pseudo-equatorial and a phenyl ring is pseudo-axial on the thia­zine ring. Both mol­ecules show a weak intra­molecular C—H⋯O inter­action between H-atom donors of one of the phenyl rings and the acetamide group. In the crystal, an inter­molecular N—H⋯O(thia­zine) hydrogen bond links *B* mol­ecules along the 2_1_ (*b*) screw axis and, in addition, an N—H⋯O(acetamide) hydrogen bond links *A* and *B* mol­ecules across *a*. A two-dimensional layered structure lying parallel to (001) is generated, also involving weak inter­molecular C—H⋯O inter­actions.

## Abstract 2

The crystal structure of the enanti­opure sulfoxide of a 2,3,5,6-tetra­hydro-1,3-thia­zin-4-one exhibits a twisted half-chair pucker for the thia­zine ring. Inter­molecular N—H ⋯O hydrogen-bonding inter­actions form a two-dimensional layered structure lying parallel to (001).

## Chemical context   

The 1,3-thia­zin-4-ones are a group of six-membered heterocycles with a wide range of biological activity (Ryabukhin *et al.*, 1996 ▸). Surrey’s research (Surrey *et al.*, 1958 ▸; Surrey, 1963*a* ▸,*b* ▸) resulted in the discovery of two drugs, the anti-anxiety and muscle relaxant chlormezanone [2-(4-chloro­phen­yl)-3-methyl-2,3,5,6-tetra­hydro-4*H*-1,3-thia­zin-4-one 1,1-dioxide] (O’Neil, 2006 ▸; Tanaka & Horayama, 2005 ▸) and muscle relaxant di­chloro­mezanone [2-(3,4-di­chloro­phen­yl)-3-methyl-2,3,5,6-tetra­hydro-4*H*-1,3-thia­zin-4-one 1,1-dioxide] (Elks & Ganellin, 1990 ▸). These sulfones showed greater activity than the sulfides from which they were synthesized (Surrey *et al.*, 1958 ▸). Surrey also prepared a variety of other sulfoxides and sulfones of 3-alkyl-2-aryl-2,3,5,6-tetra­hydro-4*H*-1,3-thia­zin-4-ones (Surrey, 1963*a* ▸,*b* ▸). We have reported previously the crystal structure of the first *N*-aryl sulfoxide in this family, racemic 2,3-diphenyl-2,3,5,6-tetra­hydro-4*H*-1,3-thia­zin-4-one 1-oxide (Yennawar *et al.*, 2016 ▸).

A sulfoxide typically has an S—O bond that is between a double bond and a single bond, with one of the lone pairs that was on the sulfide coordinating to the O atom, while O atom contributes electrons from a lone pair to a *d* orbital of the S atom. The geometry of a sulfoxide is pyramidal, with a high energy barrier for inversion, making it possible to isolate stable enanti­omers (Bentley, 2005 ▸). Herein, we report the crystal structure of the sulfoxide of *N*-[(2*S*,5*R*)-4-oxo-2,3-diphenyl-1,3-thia­zinan-5-yl]acetamide (Yennawar, Singh & Silverberg, 2015 ▸), C_18_H_18_N_2_O_3_S, prepared using the method we have reported previously for the oxidation of other 2,3-diphenyl-1,3-thia­zin-4-ones (Yennawar *et al.*, 2016 ▸; Yennawar, Noble *et al.*, 2017 ▸) and 1,3-thia­zolidinones (Yennawar, Hullihen *et al.*, 2015 ▸; Cannon *et al.*, 2015 ▸). The oxidation of the confirmed enanti­opure sulfide *N*-[(2*S*,5*R*)-4-oxo-2,3-diphenyl-1,3-thia­zinan-5-yl]acetamide 0.375-hydrate (Yennawar, Singh & Silverberg, 2015 ▸), derived from *N*-acetyl-l-cysteine, yielded a single stereoisomer as the only product.
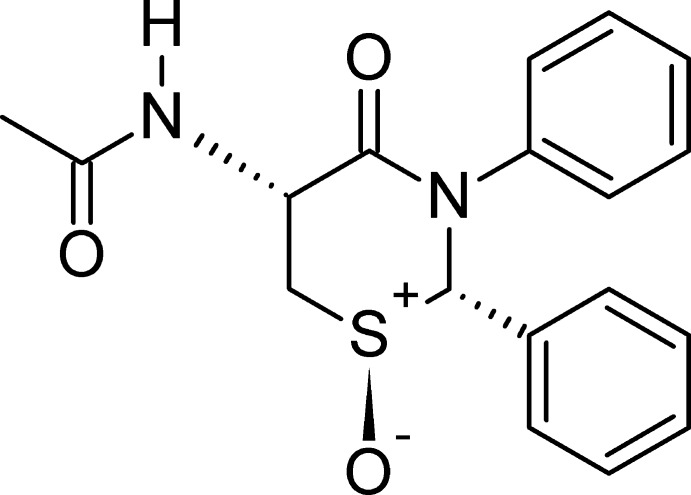


## Structural commentary   

**Figure 1:**
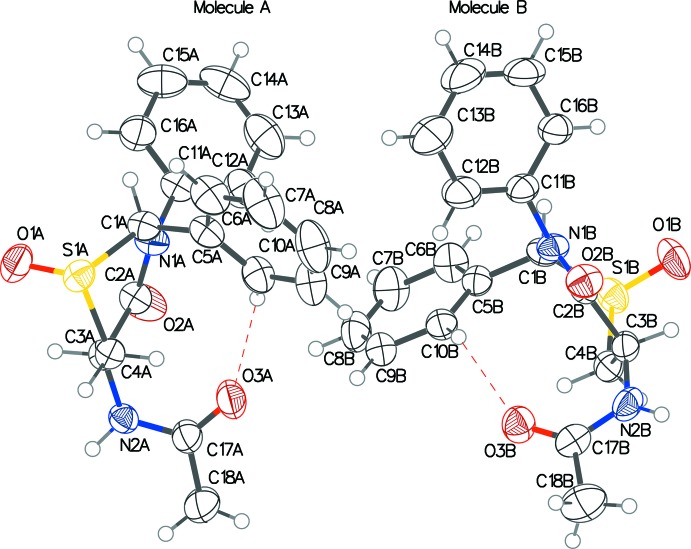
The mol­ecular structures of the two independent mol­ecules (*A* and *B*) in the asymmetric unit of the title compound, with displacement ellipsoids drawn at the 50% probability level. Dashed lines indicate intra­molecular C—H⋯O inter­actions.

The crystal structure of the title compound has two independent homochiral mol­ecules (*A* and *B*) in the asymmetric unit (Fig. 1 ▸), which have almost identical conformational features, having an alignment–r.m.s. deviation value of 0.3 Å. Both have the thia­zine rings in a twisted half-chair configuration, with puckering amplitudes = 0.6753 (19)/0.653 (2) Å and θ = 131.05 (17)/135.66 (18)° in mol­ecules *A*/*B*, respectively (Cremer & Pople, 1975 ▸). The O atom on the S atom of the ring is pseudo-axial on the thia­zine ring and *trans* to both the 2-phenyl group and the acetamide group in each case. The two phenyl rings in each mol­ecule are almost orthogonal to one another, with dihedral angles of 83.79 (17) and 86.95 (16)° in mol­ecules *A* and *B*, respectively. The acetamide group is pseudo-equatorial and the 2-phenyl group is pseudo-axial on the thia­zine ring. A weak intra­molecular C—H⋯O hydrogen bond between the 2-phenyl ring and the O atom of the acetamide group is seen in both mol­ecules (C10*A*—H⋯O3*A* and C10*B*—H⋯O3*B*), as detailed in Table 1 ▸.

We reported previously the crystal structure of the starting sulfide, *N*-[(2*S*,5*R*)-4-oxo-2,3-diphenyl-1,3-thia­zinan-5-yl]ace­t­amide 0.375-hydrate (Yennawar, Singh & Silverberg, 2015 ▸), which also had two independent homochiral mol­ecules in the asymmetric unit. However, they were not identical: in one mol­ecule, the thia­zine ring was in a half-chair conformation in which the 2-phenyl ring was nearly pseudo-axial and the acetamide group was nearly pseudo-equatorial. The other mol­ecule had the thia­zine ring in a boat conformation in which both substituents were pseudo-equatorial.

## Supra­molecular features   

**Figure 2:**
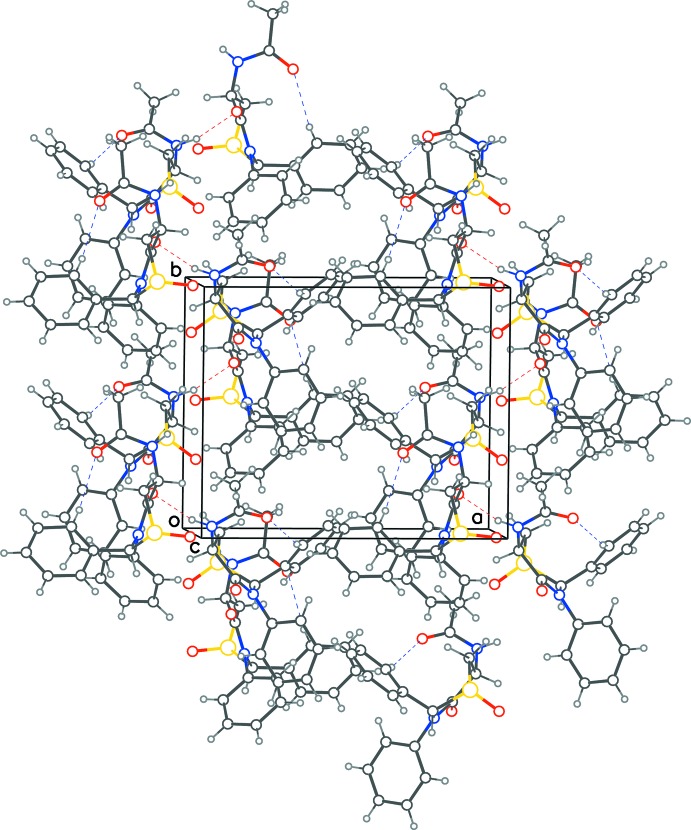
Crystal packing diagram with red dotted lines for inter­molecular N—H⋯O contacts between 2_1_-related mol­ecules, forming helical chains along the *b*-axis direction, as well as the inter­action with an independent mol­ecule. Blue dotted lines represent the intra­molecular C—H⋯O contacts.

In the crystal, the *B* mol­ecule and its 2_1_-related symmetry neighbours form a continuous hydrogen-bonded chain along the *b*-cell direction through N—H⋯O inter­actions involving the acetamide N atom and the thia­zin-1-ium-1-olate O atoms [N2*B*—H⋯O1*B* ^ii^; symmetry code: (ii) −*x*, *y* + 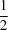, −*z*; Table 1 ▸] (Fig. 2 ▸). Mol­ecules *A* and *B* inter­act, wherein the O atom in the 4-position of mol­ecule *B* accepts a proton from the acetamide N atom of mol­ecule *A* [N2*A*—H⋯O1*B* ^i^; symmetry code: (i) *x* + 1, *y*, *z*]. The sulfoxide O atom of mol­ecule *A* does not participate in any hydrogen bonding. A two-dimensional sheet structure lying parallel to (001) is generated. No benzene ring in either of the mol­ecules participates in face-to-face π–π stacking inter­actions.

## Database survey   

Crystal structures of a number of 1,3-thia­zolidin-4-one 1-oxides have been reported (Wang *et al.*, 2010 ▸; Johnson *et al.*, 1983 ▸; Chen *et al.*, 2011 ▸; Colombo *et al.*, 2008 ▸; Yennawar, Hullihen *et al.*, 2015 ▸) and the structure of chlormezanone [2-(4-chloro­phen­yl)-3-methyl-2,3,5,6-tetra­hydro-4*H*-1,3-thia­zin-4-one 1,1-dioxide] has also been reported (Tanaka & Horayama, 2005 ▸). We have reported previously the crystal structure of 2,3-diphenyl-2,3,5,6-tetra­hydro-4*H*-1,3-thia­zin-4-one 1-oxide (Yennawar *et al.*, 2016 ▸). We have also reported recently the crystal structures of 2,3-diphenyl-2,3-di­hydro-4*H*-1,3-benzo­thia­zin-4-one 1-oxide (Yennawar, Fox *et al.*, 2017 ▸) and 2,3-diphenyl-2,3-di­hydro-4*H*-pyrido[3,2-*e*][1,3]thia­zin-4-one 1-oxide (Yennawar, Noble *et al.*, 2017 ▸).

## Synthesis and crystallization   

A 5 ml round-bottomed flask was charged with 53.9 mg of *N*-[(2*S*,5*R*)-4-oxo-2,3-diphenyl-1,3-thia­zinan-5-yl]acetamide 0.375-hydrate, whose configuration was established previously (Yennawar, Singh & Silverberg, 2015 ▸), and 1.4 ml of methanol and stirred. A solution of 79.5 mg of Oxone^®^ and 1 ml of distilled water was added dropwise and the mixture was stirred until the reaction was complete, as determined by thin-layer chromatography (TLC). The solids were dissolved by the addition of 5 ml of distilled water. The solution was extracted with 10 ml of di­chloro­methane. The organic layer was washed with 5 ml of distilled water and then with 5 ml of saturated sodium chloride. The solution was dried over Na_2_SO_4_ and concentrated under vacuum giving a crude solid. This was chromatographed on flash silica gel, eluting with a gradient of 0–60% acetone in ethyl acetate, giving 55.8 mg of product [98.6% yield; m.p. 449–452 K; *R*_F_ = 0.20 (30% acetone/70% ethyl acetate)]. Crystals suitable for X-ray crystallography were grown by slow evaporation from propan-2-ol.

## Refinement   

Crystal data, data collection and structure refinement details are summarized in Table 2 ▸. The H atoms, excepting those on N atoms, were placed geometrically and allowed to ride on their parent C atoms during refinement, with C—H distances of 0.93 (aromatic), 0.96 (meth­yl), 0.97 or (methyl­ene) and 0.98 Å (meth­yl), and with *U*_iso_(H) = 1.2*U*_eq_(aromatic or methyl­ene C) or 1.5*U*_eq_(methyl C). H atoms on N atoms were located in a difference Fourier map and were refined isotropically. The absolute configuration for the chiral centres in the mol­ecule was determined as (1*S*,2*S*,5*R*) (for the arbitrarily numbered atoms C1*A*/*B*,C3*A*/*B*), with a Flack absolute structure parameter (Flack, 1983 ▸) of 0.07 (6) for 4160 Friedel pairs.

## Supplementary Material

Crystal structure: contains datablock(s) I. DOI: 10.1107/S2056989017012488/zs2387sup1.cif

Structure factors: contains datablock(s) I. DOI: 10.1107/S2056989017012488/zs2387Isup2.hkl

Supporting information file. DOI: 10.1107/S2056989017012488/zs2387Isup3.mol

Analysis of short ring interactions.. DOI: 10.1107/S2056989017012488/zs2387sup4.pdf

CCDC reference: 1571357

Additional supporting information:  crystallographic information; 3D view; checkCIF report

## Figures and Tables

**Table 1:** Hydrogen-bond geometry (Å, °)

*D*—H⋯*A*	*D*—H	H⋯*A*	*D*⋯*A*	*D*—H⋯*A*
N2*A*—H2*A*⋯O2*B* ^i^	0.91 (3)	2.25 (3)	3.137 (3)	164 (2)
N2*B*—H2*B*⋯O1*B* ^ii^	0.79 (3)	2.14 (3)	2.916 (3)	168 (3)
C10*A*—H10*A*⋯O3*A*	0.93	2.42	3.259 (3)	149
C10*B*—H10*B*⋯O3*B*	0.93	2.44	3.232 (4)	143
C4*B*—H4*BB*⋯O2*A* ^iii^	0.97	2.25	3.116 (3)	148

Symmetry codes: (i) 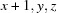; (ii) 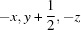; (iii) 
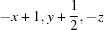
.

**Table 2:** Experimental details

Crystal data	
Chemical formula	C_18_H_18_N_2_O_3_S
*M*_r_	342.40
Crystal system, space group	Monoclinic, *P*2_1_
Temperature (K)	298
*a*, *b*, *c* (Å)	12.872 (6), 10.139 (5), 13.460 (6)
β (°)	103.104 (9)
*V* (Å^3^)	1710.8 (14)
*Z*	4
Radiation type	Mo *K*α
μ (mm^−1^)	0.21
Crystal size (mm)	0.23 × 0.20 × 0.19

Data collection	
Diffractometer	Bruker SCD area detector
Absorption correction	Multi-scan (*SADABS*; Bruker, 2016 ▸)
*T*_min_, *T*_max_	0.309, 0.900
No. of measured, independent and observed [*I* > 2σ(*I*)] reflections	15296, 8079, 6949
*R*_int_	0.031
(sin θ/λ)_max_ (Å^−1^)	0.666

Refinement	
*R*[*F* ^2^ > 2σ(*F* ^2^)], *wR*(*F* ^2^), *S*	0.049, 0.128, 1.02
No. of reflections	8079
No. of parameters	443
No. of restraints	1
H-atom treatment	H atoms treated by a mixture of independent and constrained refinement
Δρ_max_, Δρ_min_ (e Å^−3^)	0.37, −0.27
Absolute structure	Flack (1983 ▸), 4160 Friedel pairs
Absolute structure parameter	0.07 (6)

Computer programs: *SMART* (Bruker, 2016 ▸), *SAINT* (Bruker, 2016 ▸), *SHELXS97* (Sheldrick, 2008 ▸), *SHELXL97* (Sheldrick, 2008 ▸) and *OLEX2* (Dolomanov *et al.*, 2009 ▸).

## supplementary crystallographic information

### Crystal data

**Table:**

C_18_H_18_N_2_O_3_S	*D*_x_ = 1.329 Mg m^−^^3^
*M**_r_* = 342.40	Melting point = 449–452 K
Monoclinic, *P*2_1_	Mo *K*α radiation, λ = 0.71073 Å
*a* = 12.872 (6) Å	Cell parameters from 7433 reflections
*b* = 10.139 (5) Å	θ = 2.5–28.2°
*c* = 13.460 (6) Å	µ = 0.21 mm^−^^1^
β = 103.104 (9)°	*T* = 298 K
*V* = 1710.8 (14) Å^3^	Block, colorless
*Z* = 4	0.23 × 0.20 × 0.19 mm
*F*(000) = 720	

### Data collection

**Table:**

Bruker SCD area detector diffractometer	8079 independent reflections
Radiation source: fine-focus sealed tube	6949 reflections with *I* > 2σ(*I*)
Graphite monochromator	*R*_int_ = 0.031
φ and ω scans	θ_max_ = 28.2°, θ_min_ = 2.0°
Absorption correction: multi-scan (SADABS; Bruker, 2016)	*h* = −16→17
*T*_min_ = 0.309, *T*_max_ = 0.900	*k* = −13→13
15296 measured reflections	*l* = −17→17

### Refinement

**Table:**

Refinement on *F*^2^	Secondary atom site location: difference Fourier map
Least-squares matrix: full	Hydrogen site location: inferred from neighbouring sites
*R*[*F*^2^ > 2σ(*F*^2^)] = 0.049	H atoms treated by a mixture of independent and constrained refinement
*wR*(*F*^2^) = 0.128	*w* = 1/[σ^2^(*F*_o_^2^) + (0.0745*P*)^2^] where *P* = (*F*_o_^2^ + 2*F*_c_^2^)/3
*S* = 1.02	(Δ/σ)_max_ = 0.001
8079 reflections	Δρ_max_ = 0.37 e Å^−^^3^
443 parameters	Δρ_min_ = −0.27 e Å^−^^3^
1 restraint	Absolute structure: Flack (1983), 4160 Friedel pairs
Primary atom site location: structure-invariant direct methods	Absolute structure parameter: 0.07 (6)

### Special details

**Table:**

Experimental. The data collection nominally covered a full sphere of reciprocal space by a combination of 4 sets of ω scans each set at different φ and/or 2θ angles and each scan (10 s exposure) covering -0.300° degrees in ω. The crystal to detector distance was 5.82 cm.
Geometry. All esds (except the esd in the dihedral angle between two l.s. planes) are estimated using the full covariance matrix. The cell esds are taken into account individually in the estimation of esds in distances, angles and torsion angles; correlations between esds in cell parameters are only used when they are defined by crystal symmetry. An approximate (isotropic) treatment of cell esds is used for estimating esds involving l.s. planes.
Refinement. Refinement of F^2^ against ALL reflections. The weighted R-factor wR and goodness of fit S are based on F^2^, conventional R-factors R are based on F, with F set to zero for negative F^2^. The threshold expression of F^2^ > 2sigma(F^2^) is used only for calculating R-factors(gt) etc. and is not relevant to the choice of reflections for refinement. R-factors based on F^2^ are statistically about twice as large as those based on F, and R- factors based on ALL data will be even larger.

### Fractional atomic coordinates and isotropic or equivalent isotropic displacement parameters (Å^2^)

**Table:**

	*x*	*y*	*z*	*U*_iso_*/*U*_eq_	
C1A	0.79355 (19)	0.2939 (3)	0.48969 (16)	0.0397 (5)	
H1A	0.7893	0.2095	0.5241	0.048*	
C2A	0.86711 (19)	0.3246 (3)	0.33058 (17)	0.0393 (5)	
C3A	0.93950 (18)	0.4386 (2)	0.37719 (15)	0.0344 (5)	
H3A	1.0109	0.4009	0.4010	0.041*	
C4A	0.91189 (19)	0.5047 (2)	0.46957 (15)	0.0356 (5)	
H4AA	0.8426	0.5468	0.4499	0.043*	
H4AB	0.9645	0.5718	0.4964	0.043*	
C5A	0.69426 (19)	0.3706 (3)	0.49564 (17)	0.0431 (5)	
C6A	0.6572 (2)	0.3596 (4)	0.5857 (2)	0.0598 (8)	
H6A	0.6895	0.3011	0.6365	0.072*	
C7A	0.5723 (3)	0.4365 (5)	0.5981 (2)	0.0759 (11)	
H7A	0.5479	0.4297	0.6579	0.091*	
C8A	0.5243 (3)	0.5217 (5)	0.5243 (3)	0.0763 (10)	
H8A	0.4674	0.5728	0.5340	0.092*	
C9A	0.5592 (2)	0.5335 (4)	0.4343 (2)	0.0636 (8)	
H9A	0.5262	0.5926	0.3841	0.076*	
C10A	0.6433 (2)	0.4567 (3)	0.4200 (2)	0.0497 (6)	
H10A	0.6658	0.4627	0.3592	0.060*	
C11A	0.7454 (2)	0.1476 (2)	0.34101 (17)	0.0398 (5)	
C16A	0.7794 (2)	0.0238 (3)	0.3750 (2)	0.0563 (7)	
H16A	0.8400	0.0136	0.4273	0.068*	
C15A	0.7227 (3)	−0.0862 (3)	0.3309 (3)	0.0719 (10)	
H15A	0.7447	−0.1701	0.3546	0.086*	
C14A	0.6350 (3)	−0.0716 (4)	0.2531 (3)	0.0709 (10)	
H14A	0.5983	−0.1455	0.2228	0.085*	
C13A	0.6014 (3)	0.0507 (4)	0.2197 (3)	0.0734 (10)	
H13A	0.5411	0.0602	0.1671	0.088*	
C12A	0.6561 (2)	0.1616 (4)	0.2634 (2)	0.0588 (7)	
H12A	0.6325	0.2453	0.2403	0.071*	
C17A	0.8618 (2)	0.5983 (3)	0.2465 (2)	0.0487 (6)	
C18A	0.8817 (3)	0.7020 (4)	0.1734 (3)	0.0784 (11)	
H18A	0.9497	0.7427	0.1999	0.118*	
H18B	0.8266	0.7676	0.1648	0.118*	
H18C	0.8814	0.6621	0.1087	0.118*	
N1A	0.80481 (17)	0.26229 (19)	0.38641 (14)	0.0384 (4)	
N2A	0.94797 (16)	0.5352 (2)	0.29910 (14)	0.0392 (4)	
H2A	1.014 (2)	0.561 (3)	0.2912 (19)	0.036 (7)*	
O1A	1.00529 (16)	0.2968 (2)	0.56800 (15)	0.0581 (5)	
O2A	0.87065 (18)	0.2840 (2)	0.24629 (13)	0.0615 (6)	
O3A	0.77158 (16)	0.5727 (3)	0.25622 (17)	0.0702 (7)	
S1A	0.91010 (5)	0.38151 (6)	0.56562 (4)	0.04051 (15)	
C1B	0.22990 (19)	0.4488 (2)	0.06076 (17)	0.0389 (5)	
H1B	0.2075	0.3562	0.0584	0.047*	
C2B	0.16558 (17)	0.6316 (2)	0.16162 (17)	0.0357 (5)	
C3B	0.13964 (19)	0.7241 (3)	0.06778 (18)	0.0410 (5)	
H3B	0.0625	0.7164	0.0407	0.049*	
C4B	0.1904 (2)	0.6890 (3)	−0.02038 (17)	0.0418 (5)	
H4BA	0.2672	0.6983	0.0009	0.050*	
H4BB	0.1649	0.7488	−0.0769	0.050*	
C5B	0.34898 (18)	0.4469 (3)	0.06755 (16)	0.0377 (5)	
C6B	0.3913 (2)	0.3500 (3)	0.0150 (2)	0.0528 (7)	
H6B	0.3468	0.2870	−0.0229	0.063*	
C7B	0.4994 (3)	0.3473 (3)	0.0189 (2)	0.0615 (8)	
H7B	0.5269	0.2836	−0.0177	0.074*	
C8B	0.5669 (2)	0.4381 (4)	0.0764 (2)	0.0578 (7)	
H8B	0.6397	0.4350	0.0791	0.069*	
C9B	0.5264 (2)	0.5332 (3)	0.1298 (2)	0.0499 (6)	
H9B	0.5721	0.5941	0.1692	0.060*	
C10B	0.4174 (2)	0.5390 (3)	0.12537 (18)	0.0424 (5)	
H10B	0.3903	0.6043	0.1609	0.051*	
C11B	0.2058 (2)	0.4150 (2)	0.23392 (18)	0.0419 (5)	
C12B	0.2934 (3)	0.4162 (4)	0.3126 (2)	0.0722 (10)	
H12B	0.3466	0.4787	0.3143	0.087*	
C13B	0.3032 (4)	0.3236 (5)	0.3903 (3)	0.0893 (13)	
H13B	0.3635	0.3232	0.4436	0.107*	
C14B	0.2239 (3)	0.2330 (4)	0.3880 (3)	0.0775 (11)	
H14B	0.2307	0.1705	0.4397	0.093*	
C15B	0.1358 (3)	0.2339 (3)	0.3110 (3)	0.0669 (9)	
H15B	0.0818	0.1729	0.3107	0.080*	
C16B	0.1249 (2)	0.3260 (3)	0.2317 (2)	0.0505 (6)	
H16B	0.0644	0.3268	0.1787	0.061*	
C17B	0.2575 (2)	0.9072 (3)	0.13509 (19)	0.0458 (6)	
C18B	0.2671 (4)	1.0542 (3)	0.1506 (3)	0.0763 (10)	
H18D	0.3007	1.0727	0.2204	0.114*	
H18E	0.1974	1.0932	0.1342	0.114*	
H18F	0.3095	1.0903	0.1069	0.114*	
N1B	0.19754 (16)	0.5070 (2)	0.14898 (14)	0.0381 (4)	
N2B	0.15836 (19)	0.8610 (2)	0.09612 (17)	0.0469 (5)	
H2B	0.110 (2)	0.910 (3)	0.089 (2)	0.042 (8)*	
O1B	0.04145 (15)	0.5076 (3)	−0.06247 (17)	0.0666 (6)	
O2B	0.14719 (15)	0.66808 (19)	0.24289 (14)	0.0484 (4)	
O3B	0.33503 (15)	0.83467 (19)	0.15418 (15)	0.0530 (5)	
S1B	0.15756 (5)	0.52300 (7)	−0.06050 (4)	0.04663 (17)	

### Atomic displacement parameters (Å^2^)

**Table:**

	*U*^11^	*U*^22^	*U*^33^	*U*^12^	*U*^13^	*U*^23^
C1A	0.0472 (13)	0.0435 (13)	0.0292 (10)	−0.0069 (11)	0.0101 (9)	−0.0013 (9)
C2A	0.0442 (12)	0.0432 (13)	0.0316 (10)	−0.0011 (10)	0.0108 (9)	−0.0049 (9)
C3A	0.0338 (11)	0.0389 (12)	0.0300 (10)	−0.0003 (9)	0.0065 (8)	−0.0027 (9)
C4A	0.0397 (11)	0.0343 (12)	0.0319 (9)	0.0014 (9)	0.0062 (9)	−0.0056 (8)
C5A	0.0441 (12)	0.0518 (14)	0.0355 (11)	−0.0110 (12)	0.0135 (10)	−0.0064 (11)
C6A	0.0595 (16)	0.083 (2)	0.0409 (13)	−0.0101 (16)	0.0195 (12)	−0.0040 (14)
C7A	0.0571 (18)	0.125 (3)	0.0529 (16)	−0.0064 (19)	0.0280 (15)	−0.0207 (19)
C8A	0.0464 (17)	0.110 (3)	0.073 (2)	0.0079 (19)	0.0156 (15)	−0.025 (2)
C9A	0.0417 (14)	0.084 (2)	0.0621 (16)	0.0076 (16)	0.0050 (12)	−0.0074 (17)
C10A	0.0429 (13)	0.0638 (18)	0.0428 (12)	−0.0026 (13)	0.0106 (10)	−0.0038 (12)
C11A	0.0426 (13)	0.0425 (13)	0.0367 (11)	−0.0081 (11)	0.0137 (9)	−0.0063 (10)
C16A	0.0598 (17)	0.0445 (15)	0.0607 (16)	−0.0018 (14)	0.0055 (13)	−0.0077 (13)
C15A	0.100 (3)	0.0442 (18)	0.076 (2)	−0.0166 (17)	0.029 (2)	−0.0106 (14)
C14A	0.084 (2)	0.070 (2)	0.0665 (19)	−0.0399 (19)	0.0320 (19)	−0.0292 (17)
C13A	0.062 (2)	0.094 (3)	0.0601 (18)	−0.0213 (19)	0.0051 (15)	−0.0221 (18)
C12A	0.0604 (18)	0.0622 (19)	0.0468 (14)	−0.0062 (14)	−0.0028 (13)	−0.0074 (13)
C17A	0.0390 (13)	0.0625 (18)	0.0442 (13)	−0.0003 (12)	0.0085 (11)	0.0123 (12)
C18A	0.0573 (18)	0.097 (3)	0.081 (2)	0.0043 (19)	0.0149 (17)	0.050 (2)
N1A	0.0482 (11)	0.0372 (11)	0.0311 (9)	−0.0086 (9)	0.0115 (8)	−0.0076 (7)
N2A	0.0349 (10)	0.0446 (12)	0.0388 (9)	−0.0033 (9)	0.0102 (8)	0.0027 (9)
O1A	0.0572 (11)	0.0548 (12)	0.0561 (11)	0.0131 (10)	−0.0005 (9)	0.0087 (9)
O2A	0.0785 (14)	0.0743 (14)	0.0388 (9)	−0.0276 (12)	0.0280 (10)	−0.0233 (9)
O3A	0.0353 (10)	0.103 (2)	0.0692 (13)	−0.0017 (10)	0.0059 (9)	0.0341 (13)
S1A	0.0493 (3)	0.0405 (3)	0.0289 (2)	0.0010 (3)	0.0029 (2)	−0.0018 (2)
C1B	0.0428 (12)	0.0363 (12)	0.0386 (11)	−0.0073 (10)	0.0114 (10)	−0.0046 (9)
C2B	0.0284 (10)	0.0408 (12)	0.0396 (11)	−0.0031 (9)	0.0111 (9)	0.0040 (9)
C3B	0.0320 (11)	0.0487 (14)	0.0423 (12)	0.0031 (10)	0.0083 (9)	0.0088 (10)
C4B	0.0400 (12)	0.0535 (15)	0.0296 (10)	−0.0044 (11)	0.0030 (9)	0.0073 (10)
C5B	0.0390 (12)	0.0417 (13)	0.0334 (10)	0.0012 (10)	0.0099 (9)	0.0018 (9)
C6B	0.0530 (15)	0.0572 (18)	0.0474 (14)	0.0036 (13)	0.0099 (11)	−0.0136 (12)
C7B	0.0560 (16)	0.070 (2)	0.0626 (17)	0.0151 (15)	0.0223 (14)	−0.0136 (15)
C8B	0.0397 (14)	0.074 (2)	0.0604 (16)	0.0124 (14)	0.0134 (12)	0.0068 (15)
C9B	0.0410 (13)	0.0488 (15)	0.0568 (14)	−0.0004 (12)	0.0047 (11)	0.0037 (13)
C10B	0.0423 (12)	0.0381 (13)	0.0465 (12)	0.0000 (10)	0.0093 (10)	−0.0022 (10)
C11B	0.0459 (13)	0.0422 (14)	0.0414 (12)	0.0020 (10)	0.0178 (10)	0.0077 (9)
C12B	0.070 (2)	0.082 (3)	0.0588 (17)	−0.0207 (17)	0.0013 (15)	0.0301 (17)
C13B	0.096 (3)	0.104 (3)	0.061 (2)	−0.010 (2)	0.0021 (19)	0.040 (2)
C14B	0.090 (3)	0.075 (2)	0.075 (2)	0.011 (2)	0.034 (2)	0.0384 (19)
C15B	0.067 (2)	0.0498 (18)	0.097 (2)	0.0029 (15)	0.046 (2)	0.0232 (17)
C16B	0.0485 (14)	0.0436 (15)	0.0639 (17)	0.0020 (12)	0.0224 (13)	0.0070 (12)
C17B	0.0572 (16)	0.0417 (15)	0.0410 (12)	0.0062 (12)	0.0160 (11)	0.0010 (10)
C18B	0.098 (3)	0.0469 (19)	0.084 (2)	0.0031 (17)	0.021 (2)	−0.0066 (16)
N1B	0.0428 (10)	0.0384 (11)	0.0360 (9)	−0.0021 (8)	0.0148 (8)	0.0055 (8)
N2B	0.0459 (12)	0.0432 (13)	0.0541 (12)	0.0160 (11)	0.0163 (10)	0.0089 (10)
O1B	0.0388 (10)	0.0814 (16)	0.0722 (13)	−0.0158 (11)	−0.0029 (9)	−0.0075 (12)
O2B	0.0558 (11)	0.0522 (11)	0.0435 (9)	0.0023 (8)	0.0246 (8)	0.0015 (8)
O3B	0.0478 (10)	0.0490 (11)	0.0607 (11)	0.0041 (8)	0.0093 (9)	−0.0076 (8)
S1B	0.0416 (3)	0.0590 (4)	0.0362 (3)	−0.0103 (3)	0.0023 (2)	−0.0070 (3)

### Geometric parameters (Å, º)

**Table:**

C1A—H1A	0.9800	C1B—H1B	0.9800
C1A—C5A	1.514 (4)	C1B—C5B	1.515 (3)
C1A—N1A	1.465 (3)	C1B—N1B	1.468 (3)
C1A—S1A	1.841 (3)	C1B—S1B	1.846 (3)
C2A—C3A	1.527 (3)	C2B—C3B	1.547 (3)
C2A—N1A	1.371 (3)	C2B—N1B	1.351 (3)
C2A—O2A	1.217 (3)	C2B—O2B	1.227 (3)
C3A—H3A	0.9800	C3B—H3B	0.9800
C3A—C4A	1.524 (3)	C3B—C4B	1.521 (3)
C3A—N2A	1.459 (3)	C3B—N2B	1.445 (4)
C4A—H4AA	0.9700	C4B—H4BA	0.9700
C4A—H4AB	0.9700	C4B—H4BB	0.9700
C4A—S1A	1.801 (2)	C4B—S1B	1.788 (3)
C5A—C6A	1.404 (3)	C5B—C6B	1.392 (4)
C5A—C10A	1.388 (4)	C5B—C10B	1.394 (4)
C6A—H6A	0.9300	C6B—H6B	0.9300
C6A—C7A	1.383 (5)	C6B—C7B	1.380 (4)
C7A—H7A	0.9300	C7B—H7B	0.9300
C7A—C8A	1.355 (6)	C7B—C8B	1.377 (5)
C8A—H8A	0.9300	C8B—H8B	0.9300
C8A—C9A	1.389 (5)	C8B—C9B	1.374 (4)
C9A—H9A	0.9300	C9B—H9B	0.9300
C9A—C10A	1.382 (4)	C9B—C10B	1.392 (4)
C10A—H10A	0.9300	C10B—H10B	0.9300
C11A—C16A	1.372 (4)	C11B—C12B	1.361 (4)
C11A—C12A	1.375 (4)	C11B—C16B	1.372 (4)
C11A—N1A	1.449 (3)	C11B—N1B	1.460 (3)
C16A—H16A	0.9300	C12B—H12B	0.9300
C16A—C15A	1.390 (5)	C12B—C13B	1.389 (5)
C15A—H15A	0.9300	C13B—H13B	0.9300
C15A—C14A	1.362 (6)	C13B—C14B	1.369 (6)
C14A—H14A	0.9300	C14B—H14B	0.9300
C14A—C13A	1.356 (6)	C14B—C15B	1.353 (5)
C13A—H13A	0.9300	C15B—H15B	0.9300
C13A—C12A	1.385 (5)	C15B—C16B	1.401 (4)
C12A—H12A	0.9300	C16B—H16B	0.9300
C17A—C18A	1.502 (4)	C17B—C18B	1.505 (4)
C17A—N2A	1.336 (3)	C17B—N2B	1.350 (4)
C17A—O3A	1.226 (3)	C17B—O3B	1.219 (3)
C18A—H18A	0.9600	C18B—H18D	0.9600
C18A—H18B	0.9600	C18B—H18E	0.9600
C18A—H18C	0.9600	C18B—H18F	0.9600
N2A—H2A	0.91 (3)	N2B—H2B	0.79 (3)
O1A—S1A	1.491 (2)	O1B—S1B	1.497 (2)
			
C5A—C1A—H1A	106.6	C5B—C1B—H1B	106.0
C5A—C1A—S1A	108.30 (17)	C5B—C1B—S1B	111.05 (15)
N1A—C1A—H1A	106.6	N1B—C1B—H1B	106.0
N1A—C1A—C5A	115.4 (2)	N1B—C1B—C5B	115.16 (19)
N1A—C1A—S1A	112.87 (15)	N1B—C1B—S1B	111.87 (17)
S1A—C1A—H1A	106.6	S1B—C1B—H1B	106.0
N1A—C2A—C3A	120.12 (19)	N1B—C2B—C3B	118.6 (2)
O2A—C2A—C3A	119.4 (2)	O2B—C2B—C3B	119.7 (2)
O2A—C2A—N1A	120.3 (2)	O2B—C2B—N1B	121.3 (2)
C2A—C3A—H3A	106.2	C2B—C3B—H3B	105.6
C4A—C3A—C2A	115.76 (18)	C4B—C3B—C2B	116.3 (2)
C4A—C3A—H3A	106.2	C4B—C3B—H3B	105.6
N2A—C3A—C2A	110.52 (18)	N2B—C3B—C2B	112.0 (2)
N2A—C3A—H3A	106.2	N2B—C3B—H3B	105.6
N2A—C3A—C4A	111.2 (2)	N2B—C3B—C4B	110.8 (2)
C3A—C4A—H4AA	109.9	C3B—C4B—H4BA	109.7
C3A—C4A—H4AB	109.9	C3B—C4B—H4BB	109.7
C3A—C4A—S1A	108.90 (16)	C3B—C4B—S1B	109.99 (17)
H4AA—C4A—H4AB	108.3	H4BA—C4B—H4BB	108.2
S1A—C4A—H4AA	109.9	S1B—C4B—H4BA	109.7
S1A—C4A—H4AB	109.9	S1B—C4B—H4BB	109.7
C6A—C5A—C1A	117.5 (3)	C6B—C5B—C1B	119.1 (2)
C10A—C5A—C1A	123.3 (2)	C6B—C5B—C10B	119.1 (2)
C10A—C5A—C6A	119.1 (3)	C10B—C5B—C1B	121.8 (2)
C5A—C6A—H6A	120.2	C5B—C6B—H6B	119.9
C7A—C6A—C5A	119.5 (3)	C7B—C6B—C5B	120.1 (3)
C7A—C6A—H6A	120.2	C7B—C6B—H6B	119.9
C6A—C7A—H7A	119.6	C6B—C7B—H7B	119.6
C8A—C7A—C6A	120.8 (3)	C8B—C7B—C6B	120.7 (3)
C8A—C7A—H7A	119.6	C8B—C7B—H7B	119.6
C7A—C8A—H8A	119.6	C7B—C8B—H8B	120.1
C7A—C8A—C9A	120.7 (3)	C9B—C8B—C7B	119.8 (3)
C9A—C8A—H8A	119.6	C9B—C8B—H8B	120.1
C8A—C9A—H9A	120.3	C8B—C9B—H9B	119.8
C10A—C9A—C8A	119.4 (3)	C8B—C9B—C10B	120.4 (3)
C10A—C9A—H9A	120.3	C10B—C9B—H9B	119.8
C5A—C10A—H10A	119.8	C5B—C10B—H10B	120.0
C9A—C10A—C5A	120.5 (3)	C9B—C10B—C5B	119.9 (2)
C9A—C10A—H10A	119.8	C9B—C10B—H10B	120.0
C16A—C11A—C12A	119.8 (3)	C12B—C11B—C16B	120.9 (3)
C16A—C11A—N1A	119.7 (2)	C12B—C11B—N1B	120.3 (2)
C12A—C11A—N1A	120.5 (3)	C16B—C11B—N1B	118.8 (2)
C11A—C16A—H16A	120.2	C11B—C12B—H12B	120.1
C11A—C16A—C15A	119.6 (3)	C11B—C12B—C13B	119.9 (3)
C15A—C16A—H16A	120.2	C13B—C12B—H12B	120.1
C16A—C15A—H15A	119.9	C12B—C13B—H13B	120.1
C14A—C15A—C16A	120.3 (3)	C14B—C13B—C12B	119.8 (4)
C14A—C15A—H15A	119.9	C14B—C13B—H13B	120.1
C15A—C14A—H14A	120.0	C13B—C14B—H14B	119.9
C13A—C14A—C15A	120.0 (3)	C15B—C14B—C13B	120.3 (3)
C13A—C14A—H14A	120.0	C15B—C14B—H14B	119.9
C14A—C13A—H13A	119.7	C14B—C15B—H15B	119.7
C14A—C13A—C12A	120.6 (3)	C14B—C15B—C16B	120.7 (3)
C12A—C13A—H13A	119.7	C16B—C15B—H15B	119.7
C11A—C12A—C13A	119.7 (3)	C11B—C16B—C15B	118.5 (3)
C11A—C12A—H12A	120.2	C11B—C16B—H16B	120.7
C13A—C12A—H12A	120.2	C15B—C16B—H16B	120.7
N2A—C17A—C18A	116.0 (2)	N2B—C17B—C18B	116.0 (3)
O3A—C17A—C18A	121.6 (3)	O3B—C17B—C18B	122.0 (3)
O3A—C17A—N2A	122.4 (2)	O3B—C17B—N2B	121.9 (2)
C17A—C18A—H18A	109.5	C17B—C18B—H18D	109.5
C17A—C18A—H18B	109.5	C17B—C18B—H18E	109.5
C17A—C18A—H18C	109.5	C17B—C18B—H18F	109.5
H18A—C18A—H18B	109.5	H18D—C18B—H18E	109.5
H18A—C18A—H18C	109.5	H18D—C18B—H18F	109.5
H18B—C18A—H18C	109.5	H18E—C18B—H18F	109.5
C2A—N1A—C1A	127.96 (19)	C2B—N1B—C1B	128.89 (19)
C2A—N1A—C11A	117.19 (18)	C2B—N1B—C11B	117.93 (19)
C11A—N1A—C1A	114.79 (18)	C11B—N1B—C1B	113.1 (2)
C3A—N2A—H2A	120.0 (16)	C3B—N2B—H2B	120 (2)
C17A—N2A—C3A	121.0 (2)	C17B—N2B—C3B	121.5 (2)
C17A—N2A—H2A	118.6 (16)	C17B—N2B—H2B	119 (2)
C4A—S1A—C1A	94.46 (11)	C4B—S1B—C1B	94.54 (11)
O1A—S1A—C1A	107.22 (13)	O1B—S1B—C1B	105.93 (12)
O1A—S1A—C4A	105.71 (11)	O1B—S1B—C4B	105.70 (14)

### Hydrogen-bond geometry (Å, º)

**Table:**

*D*—H···*A*	*D*—H	H···*A*	*D*···*A*	*D*—H···*A*
N2*A*—H2*A*···O2*B*^i^	0.91 (3)	2.25 (3)	3.137 (3)	164 (2)
N2*B*—H2*B*···O1*B*^ii^	0.79 (3)	2.14 (3)	2.916 (3)	168 (3)
C10*A*—H10*A*···O3*A*	0.93	2.42	3.259 (3)	149
C10*B*—H10*B*···O3*B*	0.93	2.44	3.232 (4)	143
C4*B*—H4*BB*···O2*A*^iii^	0.97	2.25	3.116 (3)	148

Symmetry codes: (i) *x*+1, *y*, *z*; (ii) −*x*, *y*+1/2, −*z*; (iii) −*x*+1, *y*+1/2, −*z*.
