# Supplementary material for: Crystal structure of (1S,2S,5R)-5-acetyl­amino-4-oxo-2,3-diphenyl-1,3-thia­zinan-1-ium-1-olate
Source: Acta Crystallogr E Crystallogr Commun. 2017 Sep 5;73(Pt 10):1417–20. doi: 10.1107/S2056989017012488 (PMC5730286; doi:10.1107/S2056989017012488)
Supplement: Supplementary file 4 [file e-73-01417-sup4.pdf]

Analysis of Short Ring-Interactions with Cg-Cg Distances <   6.0 Angstrom and Beta < 60.0Deg.

===

- Cg(I)   = Plane number I (= ring number in () above)
- Alpha   = Dihedral Angle between Planes I and J (Deg)
- Beta   = Angle Cg(I)-->Cg(J) or Cg(I)-->Me vector and normal to plane I (Deg)
- Gamma   = Angle Cg(I)-->Cg(J) vector and normal to plane J (Deg)
- Cg-Cg   = Distance between ring Centroids (Ang.)
- CgI_Perp = Perpendicular distance of Cg(I) on ring J (Ang.)
- CgJ_Perp = Perpendicular distance of Cg(J) on ring I (Ang.)
- Slippage = Distance between Cg(I) and Perpendicular Projection of Cg(J) on Ring I (Ang).
- P,Q,R,S = J-Plane Parameters for Carth. Coord. (Xo, Yo, Zo)

Cg(I)	Cg(J)	Cg-Cg	Alpha	Beta	Gamma	CgI_Perp	CgJ_Perp
Cg(2)	-> Cg(6)[i]	5.313(3)	69.87(17)	33.1	84.8	-0.4825(14)	-4.4494(15)
Cg(2)	-> Cg(6)[ii]	4.818(3)	55.39(17)	12.8	62.4	-2.2318(14)	4.6984(15)
Cg(3)	-> Cg(2)[iii]	5.186(3)	80.36(17)	17.5	81.7	-0.7521(14)	-4.9464(12)
Cg(3)	-> Cg(5)[iv]	4.999(3)	53.60(15)	57.5	28.6	-4.3887(14)	2.6896(11)

Ring(2): C5-C10   Ring(3): C11-C16   Ring(5): C23-C28   Ring(6): C29-C34

Symmetry codes: [i] = X,Y,Z   [ii] = -1-X,1/2+Y,-1-Z   [iii] = -1-X,-1/2+Y,-1-Z   [iv] = -1-X,-1/2+Y,-2-Z
